# Supplementary material for: Mechanochemical-Assisted Extraction and Hepatoprotective Activity Research of Flavonoids from Sea Buckthorn (Hippophaë rhamnoides L.) Pomaces
Source: Molecules. 2021 Dec 15;26(24):7615. doi: 10.3390/molecules26247615 (PMC8704866; doi:10.3390/molecules26247615)
Supplement: Supplementary file 1 [file molecules-26-07615-s001.zip › molecules-1460559-supplementary.pdf]

Supplementary Materials

Mechanochemical-Assisted Extraction and Hepatoprotective Activity Research of Flavonoids from Sea Buckthorn (*Hippophaë rhamnoides* L.) Pomaces

Zili Guo, Jingya Cheng, Lei Zheng, Wenhao Xu * and Yuanyuan Xie *

Collaborative Innovation Center of Yangtze River Delta Region Green Pharmaceuticals, College of Pharmaceutical Sciences, Zhejiang University of Technology, Hangzhou, 31000, China;
guozili@zjut.edu.cn (Z.G.); chengjy@zjut.edu.cn (J.C.); 18267735533@163.com (L.Z.)
* Correspondence: xuwenhao@zjut.edu.cn (W.X.); xyycz@zjut.edu.cn (Y.X.); Tel.: +86-189-5805-7635 (W.X.) & (Y.X.)

Table S1. Box-Behnken experiment design with the independent variables.

Run	X_1	X_2	X_3	Yield of flavonoids (mg/g)
1	500	10	30：1	20.85
2	500	20	40：1	22.63
3	400	30	20：1	23.72
4	400	20	30：1	26.73
5	500	30	30：1	25.43
6	400	30	40：1	23.91
7	400	10	20：1	20.78
8	400	20	30：1	27.63
9	400	10	40：1	18.94
10	300	30	30：1	23.92
11	400	20	30：1	25.83
12	300	10	30：1	21.34
13	300	20	20：1	23.75
14	400	20	30：1	27.18
15	500	20	20：1	23.21
16	300	20	40：1	22.39
17	400	20	30：1	26.28

Table S2. Regression model significance and analysis of variance.

Source	df	Sum of squares	Mean square	F-value	P-value
Model	9	96.52	10.72	32.31	<0.0001**
X_1	1	0.065	0.065	0.20	0.6719
X_2	1	28.39	28.39	85.52	<0.0001**
X_3	1	1.61	1.61	4.85	0.0634
X_1X_2	1	1.00	1.00	3.01	0.1262
X_1X_3	1	0.15	0.15	0.46	0.5202
X_2X_3	1	1.03	1.03	3.10	0.1121
X_1^2	1	7.60	7.60	22.90	0.0020
X_2^2	1	26.34	25.34	79.36	0.0075**
X_3^2	1	24.08	24.08	72.53	<0.0001**
Residual	7	2.32	0.33		<0.0001**
Lack of Fit	3	0.30	0.10	0.20	
Pure Error	4	2.02	0.51		0.8937
Cor Total	16	98.84	R^2= 0.9765		

Note: df, degree of freedom. * $p < 0.05$ significant. ** $p < 0.01$ highly significant.

Table S3. Effect of NAFLD, curcumin, and sea buckthorn pomaces extracts on biochemical parameters in tetracycline liver cirrhosis mice.

Animal group	Serum				AST	ALT	Liver			
	TG	TC	HDL-C	LDL-C			TG	TC	HDL-C	LDL-C
NCG	1.76 ± 0.10	1.81 ± 0.11	0.82 ± 0.15	0.42 ± 0.08	59.91 ± 3.40	51.67 ± 2.30	0.28 ± 0.09	0.54 ± 0.10	2.13 ± 0.45	0.17 ± 0.08
NMG	2.28 ± 0.10	3.62 ± 0.09	4.11 ± 0.31	10.18 ± 1.68	106.21 ± 6.22	128.17 ± 7.37	0.74 ± 0.15	1.17 ± 0.35	0.47 ± 0.28	0.22 ± 0.04
MPG 200 mg/kg	2.02 ± 0.11	2.70 ± 0.07	2.39 ± 0.07	8.83 ± 0.89	74.89 ± 11.39	61.65 ± 3.78	0.49 ± 0.09	0.86 ± 0.22	1.06 ± 0.15	0.13 ± 0.09
HPG 200 mg/kg	2.01 ± 0.16	2.11 ± 0.13	2.16 ± 0.21	8.05 ± 1.32	70.12 ± 2.86	72.27 ± 6.08	0.66 ± 0.12	1.11 ± 0.18	0.71 ± 0.40	0.20 ± 0.12
CCG 200 mg/kg	2.06 ± 0.09	2.09 ± 0.03	2.24 ± 0.11	7.78 ± 1.36	82.51 ± 3.58	59.26 ± 3.88	0.54 ± 0.07	0.96 ± 0.20	1.03 ± 0.51	0.21 ± 0.06

All values are expressed as mean ± SEM. of eight mice in each group. TG: triglycerides, TC: total cholesterol, HDL-C: high-density lipoprotein, LDL-C: low-density lipoprotein, ALT: alanine aminotransferase, AST: aspartate aminotransferase.

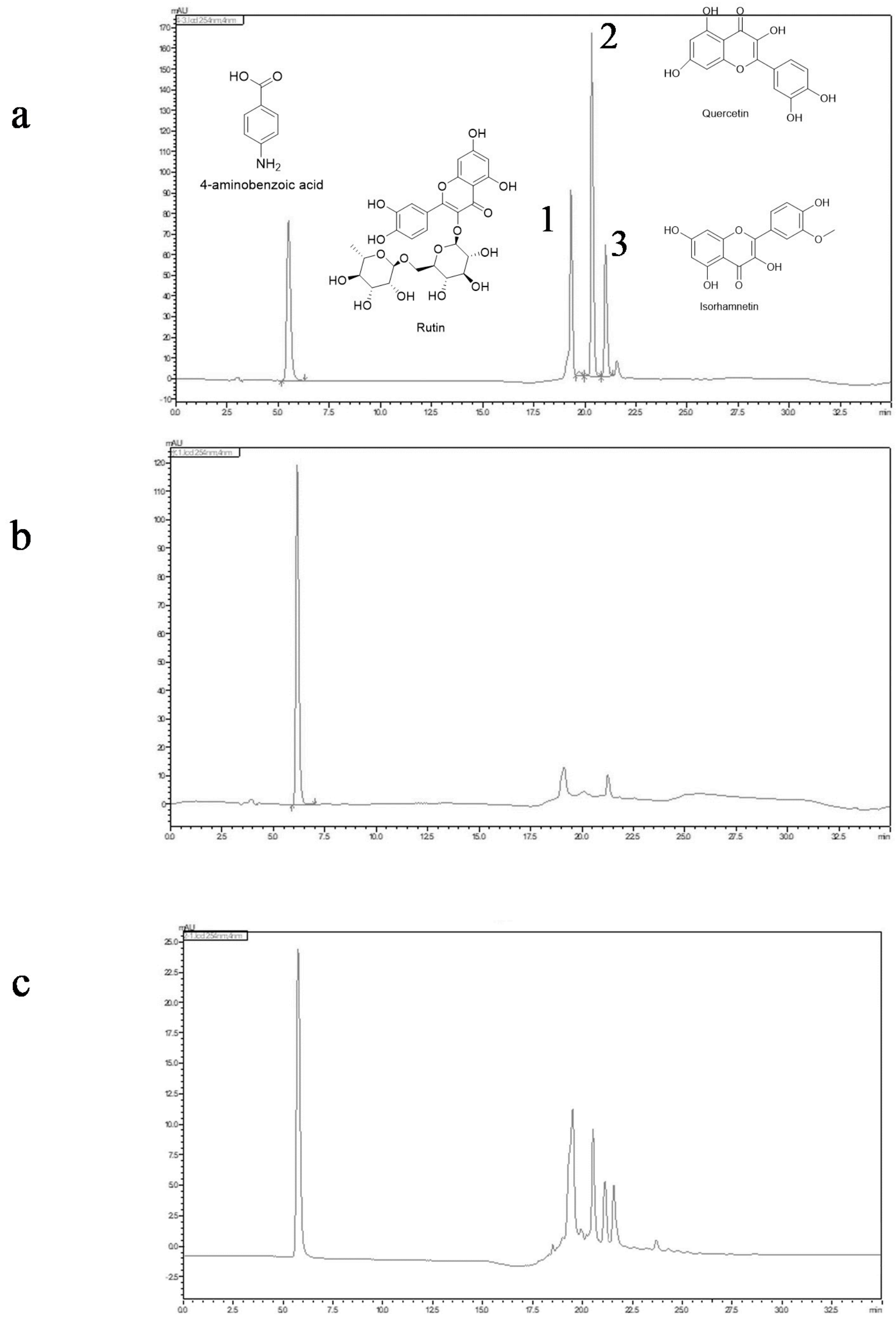

Figure S1. HPLC analysis results: (a) internal standard and references; (b) the HPLC diagram of sea buckthorn flavonoids extracted by HRE method (HPG); (c) the HPLC diagram of sea buckthorn flavonoids extracted by MCAE method (MPG).
